# Dr. S. S. Satchwell

**Published:** 1873-04

**Authors:** 


*Dr. S. S. Satchwell.*

This distinguished medical gentleman of North Carolina, and one of our ablest associate editors, delivered recently an address before the "Alumni Association of the University of the City of New York," which has been eulogized for its learning and beauty of diction by the press of New York city. Dr. Satchwell is worthy of the honors so fraternally bestowed upon him by the profession and press of that city, and we trust this just and public acknowledgment of the distinguished merit of one of our Southern medical brethren will serve as an additional link in the chain of fraternal kindness in binding more closely the brotherhood of the two geographical sections.

Dr. Satchwell's reputation is not confined to the good old State of North Carolina—where he has attained the highest professional distinction in the gift of his medical brethren—but is known as an author and essayist throughout the country.

As one of our editorial staff, we appreciate and value the high degree of respect and compliments bestowed on our associate—thanking our Northern brethren for the courtesy they have shown him, and the opportunity thus given us of testifying to his worth and ability.

